# Global scientific trends on aflatoxin research during 1998–2017: a bibliometric and visualized study

**DOI:** 10.1186/s12995-019-0248-7

**Published:** 2019-11-21

**Authors:** Sa’ed H. Zyoud

**Affiliations:** 1 Poison Control and Drug Information Center (PCDIC), College of Medicine and Health Sciences, An-Najah National University, Nablus, 44839 Palestine; 2 Department of Clinical and Community Pharmacy, College of Medicine and Health Sciences, An-Najah National University, Nablus, 44839 Palestine; 3 Clinical Research Centre, An-Najah National University Hospital, Nablus, 44839 Palestine

**Keywords:** Aflatoxin, Scopus, Bibliometric, VOSviewer

## Abstract

**Background:**

Aflatoxins are fungal metabolites associated with contaminated food products. Intake of aflatoxin-contaminated food results in serious health hazards and even death. Therefore, the aim of this study is to evaluate the global scientific output of research of aflatoxin by using bibliometric techniques.

**Methods:**

This bibliometric study was conducted using Scopus database and classified the retrieved publications were classified from different aspects, including the countries/region of focus, journals, authors, institutes, citations, and content analysis to discover any hot and emerging topics. In addition, the bibliometric analysis of the international collaborative network and hot research topics were generated by VOSviewer© software version 1.6.10. The publication period was restricted in the search for two decades (1998–2017).

**Results:**

The search engine of the Scopus database found 9845 documents published in the field of aflatoxin. The USA is the top publishing source in the world (22.85%), followed by China (11.85%), India (9.32%), and Italy (5.25%). In earlier years, researchers focused on terms related to the topics of “sources and biosynthesis of aflatoxin”, “health effects by aflatoxin”, and “detoxification and treatment of aflatoxin”. However, in recent years, researchers pay more attention to the topic of detection and quantification of aflatoxin.

**Conclusions:**

The quantity of research in global aflatoxin has substantially increased over the past two decades. The evaluation of the historical status and development trend in aflatoxin scientific research can guide future research, and ultimately provide the basis for improving management procedures for governmental decisions, healthcare, industries, and educational institutions.

## Background

Aflatoxins are toxic secondary metabolites, affected by fungal species, of Aspergillus molds that are largely distributed in nature and have contaminated the food supplies of animals and humans, resulting in serious health hazards and even death [1, 2]. Additional health impacts of aflatoxins include hepatotoxicity, teratogenicity, genotoxicity, and cytotoxicity [3]. It has been estimated about 5 billion people globally are particularly affected by exposed to dietary aflatoxins [4]. Tropical and subtropical areas of the world are the highest areas for aflatoxin contamination of the food products, where food storage conditions for cereals (e.g. maize and peanuts), spices, and milk are suboptimal [4–6].

Bibliometrics and evaluation of research performance have been carried out on a wide range of health topics [7–15], and several have been carried out in the fields of environmental studies [16, 17], and toxicology [18–24]. Yet, to the best of my knowledge, only a few bibliometric studies in food contamination have been done recently [25–28], and only one bibliometric study explicitly focused on aflatoxin has been published by using Web of Science (WoS) database for data collection [28]. Because the aflatoxin bibliometric study [28] found that aflatoxin research is now being given increased scientific attention internationally, it is therefore necessary to thoroughly evaluate and classify the existing literature from different aspects, including the countries/region of origin, journals, authors, institutes, citations, and content analysis to discover any hot and emerging topics using a large and comprehensive database. Therefore, the aim of this study is to evaluate the global scientific output of research of aflatoxin by using bibliometric techniques, and flag areas of concern.

Identifying the most productive and influential research, can be useful to anyone involved on the field of aflatoxin. Drawing on these insights may aid understanding of historical progress in aflatoxin research over the last 20 years and offer guidance researchers, and policy makers, regarding best scientific and publishing practices for future health research of this scientific field.

## Methods

### Data source

In this bibliometric study, we selected documents related to aflatoxin indexed in Scopus database from 1998 to 2017. This database is the large one, when compared with PubMed or Web of Science, and usually respected as a reliable source for academic and bibliometric studies [29, 30]. The use of Scopus as a bibliometric tool was based on the idea that it has a better coverage of journals than other databases such as Web of Science [29, 30]. Additionally, Scopus has been used and validated in previously published bibliometric analyses [8, 9, 18, 31–37]. Data were collected in March 2019.

### Search strategy

The following search string was used to identify publications in the field of aflatoxin based on their titles and/or abstract: TITLE-ABS (aflatox*) AND PUBYEAR >1997 AND PUBYEAR <2018. To get greater accuracy in the findings, the search strategy for the terms related to aflatoxin was limited to Title/Abstract only because if expanded to other search fields such Keywords, many publications identified were not related to aflatoxin (i.e false-positive data). Researchers’ experience [7, 35, 38, 39] is that inclusion of search items in the title/abstract instead of a topic search (title, abstract, and keywords greatly increases specificity with minimum loss of sensitivity. The major reason for the generation of false-positive results by keyword search is that Scopus considers Keywords as author and indexed keywords such as “EMTRE drug terms”, “EMTREE medical terms”, and “Medline keywords”.

### Bibliometric analysis

The evaluation of the collected sample involved weighing the following indictors: (1) publication output by years, (2) top 10 countries with their h-index and collaboration pattern, (3) top 10 most influential journals with their Source Normalized Impact per Paper (SNIP), and impact factors (IF), (4) top 10 most influential institutions, and (5) top 20 cited publications.

### Visualized analysis

The bibliometric analysis of the international collaborative network and hot research topics were generated by VOSviewer© software version 1.6.10 [40]. This freely available computer program (www.vosviewer.com) that is used for constructing and viewing bibliometric maps to analyze the output of countries, and authors in this sphere, and it highlights commonly used terms in the titles and abstracts for the retrieved publications, revealing those hot research topics.

## Results and discussion

**Fig. 1:**
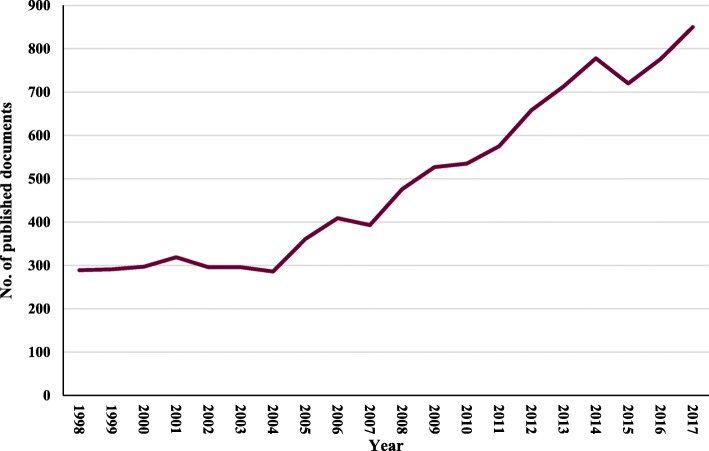
Number of publications per year (1998–2017)

The search engine of the Scopus database found 18,342 documents published in the field of aflatoxin from 1963 to 2018 (Additional file 1). The first publications appeared in 1963 [41–43]. After this, the number of publications grew gradually and slowly each year, with little fluctuation (Additional file 1). Of them, 9845 documents published in the field of aflatoxin from 1998 to 2017. Within this batch, this study juggled 8288 articles, 687 reviews, and 870 other types of documents, including letters, article end notes, editorials, and minutes of meetings. Figure 1 shows the publication trend related to aflatoxin from 1998 to 2017. The results reveal that the number of annual publications had gradually increased during 1998–2004, indicating that research output showed steady growth during those years. Prominently, the number of relevant publications increased sharply since 2004; meanwhile, 2017 netted the largest haul of aflatoxin research (850 documents published). The data indicates indeed that issues related to aflatoxin are becoming increasingly important in the investigation of food safety and human health. English is the predominant language of publications on aflatoxin, constituting 93.8% of the total, with only 6.2% of the publications in another language. The most common non-English language is Chinese, which constitutes 2.1% of the total, followed by Portuguese (0.8%). The reason for this finding is that Scopus has a better coverage of English language journal than those in other languages [44]. A previously published study on aflatoxin [28] had shown different results (5122 documents worldwide from 1963 to 2016) from those presented in the current study. The difference was due to (1) different databases used to retrieve the documents and (2) research domains being investigated. The study by Klingelhöfer et al. [28] was conducted using WoS and was limited to biomedical research areas. In the current study, Scopus database was used without limiting the results to any particular research area.

The top 10 countries of origin are shown in Table 1, which published 7348 documents (74.63%) of all publications. The USA is the leader (22.85%), followed by China (11.85%), India (9.32%), and Italy (5.25%). Consistent with other previous bibliometric studies [20, 21, 23, 28, 45], most of the publications in the field of aflatoxin research were published in the United States. As the USA is at the forefront of scientific research and academics, and hence, this trend is expected and in line with other bibliometrics [46]. A possible explanation for these results may be due to large part of efforts by the Maryland-based National Institutes of Health (NIH) funding for aflatoxin research, which was granted authority when several outbreaks of human illness caused by aflatoxins had been reported in some developing countries [47–51]. China is the second prolific producer in this field with 11.85% of the world total publications. It seems possible that these results are due to large part of efforts by the Beijing-headquartered National Natural Science Foundation of China (NSFC) funding for aflatoxin research [52]. Hence, Chinese authorities might be responding to the emerging problems arising from the rises in deaths from hepatocellular carcinoma which seem related to an increase in aflatoxin contamination of Chinese staple foods and this might have made Aflatoxin research a high and growing priority in China [53].

**Table 1:** Top 10 most productive countries for aflatoxin research

Ranking	Country	Number of publications (%)	h-index	No of collaboration countries	No of documents from collaboration
1st	United States	2250 (22.85)	118	87	810
2nd	China	1167 (11.85)	67	43	284
3rd	India	918 (9.32)	54	46	126
4th	Italy	517 (5.25)	62	54	180
5th	Iran	505 (5.13)	40	25	73
6th	Brazil	494 (5.02)	46	40	145
7th	Turkey	442 (4.49)	44	20	44
8th	United Kingdom	372 (3.78)	66	63	240
9th	Egypt	364 (3.70)	39	36	143
10th	Japan	319 (3.24)	49	33	120

Among the top 10 countries, five (i.e. India, Iran, Brazil, Turkey, and Egypt) were developing countries as defined by the United Nations, which suggests that each perceive this issue as a serious problem. Among this grouping, there was international diversity not associated with the traditional researching nations’ scientific productivity ranking [18, 34–36, 54–56]. The current data verified that Turkey, Egypt and Iran have been the main research contributors from the Middle Eastern countries. Consecutive outbreaks of acute aflatoxicosis in developing countries [4, 6, 57] (specifically, Turkey [58, 59], Iran [60], India [61–64], Brazil [51, 65], and Egypt [66]) caused exceptionally large morbidity and mortality connected with such outbreaks [1, 67–69], and this may explain why more research has emphasized on aflatoxin since that time [28].

**Fig. 2:**
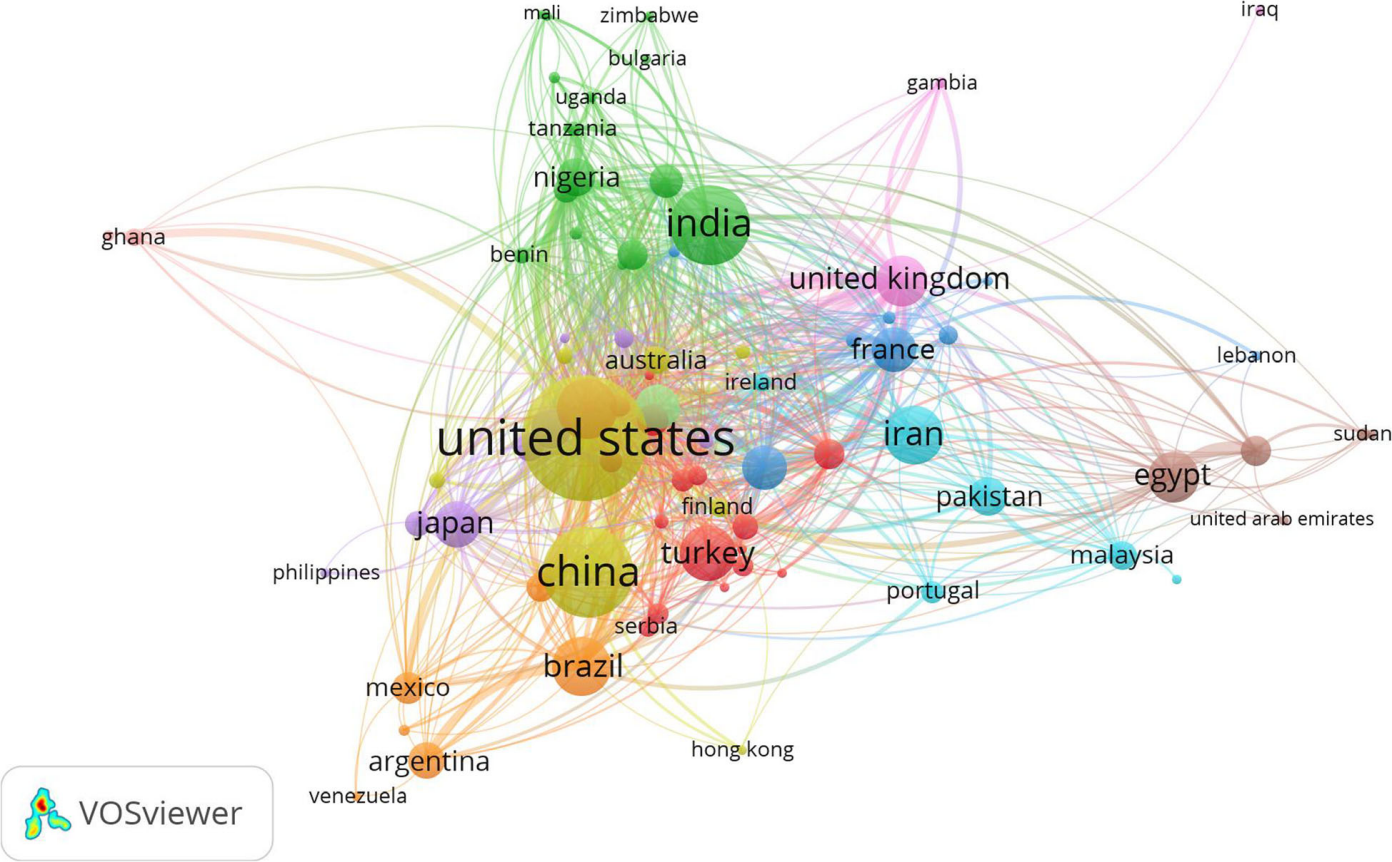
Network visualization map for country collaboration. The minimum number of documents of an author was 10. 80 countries meet this threshold as illustrated in 11 clusters. Countries represented with larger circle size or font size had relatively more publications

Analysis of international collaborations (i.e., link strength) showed that the United States had the highest number of collaborators (*n* = 87) followed by the United Kingdom (*n* = 63), and Italy (*n* = 54) (Fig. 2 and Table 1). The h-index, or Hirsch index, has been demonstrated for each country in Table 1, and it is a measure that combines both the productivity (number of publications) and their citations (perceived as an index of research quality) [70].

**Fig. 3:**
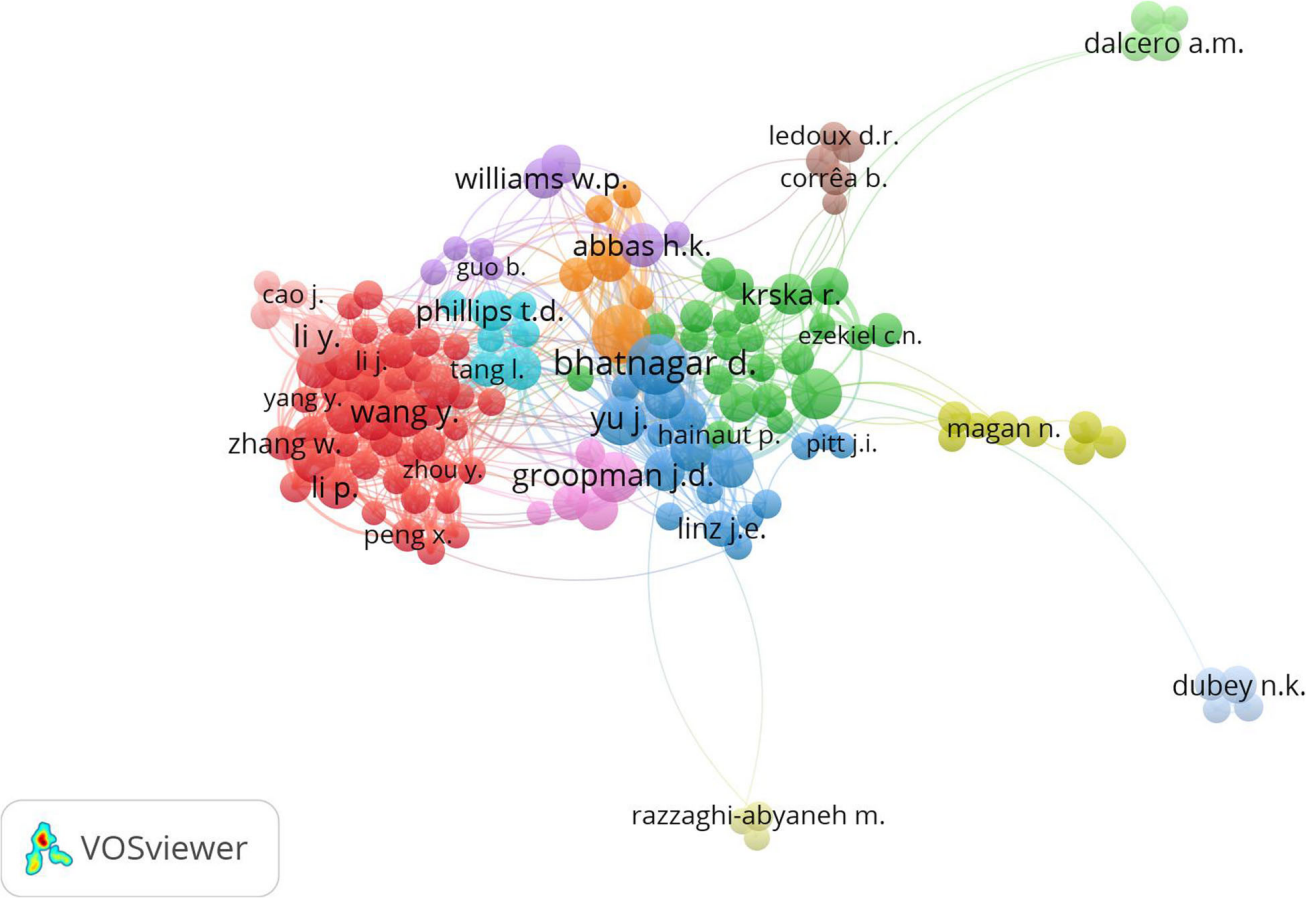
Network visualization map for author collaboration. The minimum number of documents of an author was 20. Of 23,224 authors, 149 meet this threshold as illustrated in 13 clusters. Authors represented with larger circle size or font size had relatively more publications

Figure 3 illustrates the network visualization map for author collaboration, showing 149 authors with more than 20 documents published. Approximately 23,224 unique authors participated in publishing the retrieved publications, an average of 2.36 authors per publication. D. Bhatnagar was the most active author with 118 publications. The top 10 journals that published on this topic are listed in Table 2. *Food Control* published the highest number of articles (384, 3.90%), followed by *Food and Chemical Toxicology* (158, 1.60%) and *Toxins* (158, 1.60%). The top 10 journals with the greatest contribution to aflatoxin research accounted for 16.70% of all publications included in this study.

**Table 2:** Most influential journals publishing aflatoxin research

Ranking^a^	Journal	Number of publications (%)	IF^b^	SNIP^c^
1st	*Food Control*	384 (3.90)	4.248	1.731
2nd	*Food and Chemical Toxicology*	158 (1.60)	3.775	1.277
2nd	*Toxins*	158 (1.60)	3.895	1.245
4th	*Mycotoxin Research*	151 (1.53)	3.741	1.187
5th	*Food Additives and Contaminants: Part A*	146 (1.48)	2.170	0.909
5th	*International Journal of Food Microbiology*	146 (1.48)	4.006	1.556
7th	*Journal of Agricultural and Food Chemistry*	143 (1.45)	3.571	1.321
8th	*World Mycotoxin Journal*	140 (1.42)	2.406	0.840
9th	*Journal of Food Protection*	115 (1.17)	1.559	0.744
10th	*Food Additives and Contaminants*^*d*^	103 (1.05)	NA	1.355

*SNIP* Source Normalized Impact per Paper, *IF* Impact factor, *NA* Not available

^a^Equal journals have the same ranking number, and then a gap is left in the ranking numbers

^b^Impact factors (IF) based on Journal Citation Reports (JCR) 2018 from Clarivate Analytics

^c^SNIP based on Scopus data which was freely available at www.scopus.com/sources

^d^Continued as: Food Additives & Contaminants: Part A (2008 - current), and Food Additives & Contaminants: Part B: Surveillance (2008 - current)

The analysis of the 20 most cited publications in the last 2 decades (Table 3) revealed that there is no close relationship between the number of citations from a specific publication and the most active journals in the area. HS Hussein and JM Brasel’s “Toxicity, metabolism, and impact of mycotoxins on humans and animals” published in 2001 in the journal *Toxicology* is considered the most highly cited aflatoxin piece in all of Scopus. The most cited article on aflatoxin was published by *Toxicology*, which was not listed in the top 10 journals. Characteristics of the top 20 most-cited publications on aflatoxin [5, 71–89] are presented in Table 3. Although it is difficult to demonstrate the quality or influence of publications by bibliometric analysis, the number of article citations can indicate the value and significance of the journal to some extent [90]. In addition, such analyses for the top 20 most-cited titles can help guide researchers and toxicologists towards up-to-date knowledge of the current trends in basic research, the changing landscape in food safety, and significant future research directions [91].

**Table 3:** Top-cited papers in the Journal from 1998 through 2017 according to the number of citations in Scopus

Rank	Authors	Title	Year	Source title	Cited by	Document type
1st	Hussein and Brasel [71]	“Toxicity, metabolism, and impact of mycotoxins on humans and animals”	2001	Toxicology	868	Review
2nd	Williams et al. [5]	“Human aflatoxicosis in developing countries: A review” of toxicology, exposure, potential health consequences, and interventions”	2004	American Journal of Clinical Nutrition	822	Review
3rd	Bosch et al. [72]	“Epidemiology of primary liver cancer”	1999	Seminars in Liver Disease	796	Article
4th	Machida et al. [73]	“Genome sequencing and analysis of Aspergillus oryzae”	2005	Nature	747	Article
5th	Creppy [74]	“Update of survey, regulation and toxic effects of mycotoxins in Europe”	2002	Toxicology Letters	692	Conference Paper
6th	Bosch et al. [75]	“Epidemiology of hepatocellular carcinoma”	2005	Clinics in Liver Disease	653	Conference Paper
7th	Placinta et al. [76]	“A review of worldwide contamination of cereal grains and animal feed with Fusarium mycotoxins”	1999	Animal Feed Science and Technology	619	Article
8th	Lunn et al. [77]	“XRCC1 polymorphisms: effects on aflatoxin B1-DNA adducts and glycophorin A variant frequency”	1999	Cancer Research	513	Article
9th	Okuda [78]	“Hepatocellular carcinoma”	2000	Journal of Hepatology	510	Article
10th	Whittaker et al. [79]	“The role of signaling pathways in the development and treatment of hepatocellular carcinoma”	2010	Oncogene	506	Article
11th	El-Serag [80]	“Hepatocellular carcinoma: An epidemiologic view”	2002	Journal of Clinical Gastroenterology	501	Conference Paper
12th	Richard [81]	“Some major mycotoxins and their mycotoxicoses-An overview”	2007	International Journal of Food Microbiology	475	Article
13th	Yu et al. [82]	“Clustered Pathway Genes in Aflatoxin Biosynthesis”	2004	Applied and Environmental Microbiology	453	Short Survey
14th	Turner et al. [83]	“Analytical methods for determination of mycotoxins: A review”	2009	Analytica Chimica Acta	447	Review
15th	D’Mello et al. [84]	“Fusarium mycotoxins: A review of global implications for animal health, welfare and productivity”	1999	Animal Feed Science and Technology	433	Article
16th	McMahon [85]	“The natural history of chronic hepatitis B virus infection”	2009	Hepatology	423	Article
17th	Peraica et al. [86]	“Toxic effects of mycotoxins in humans”	1999	Bulletin of the World Health Organization	414	Article
18th	Gomaa et al. [87]	“Hepatocellular carcinoma: Epidemiology, risk factors and pathogenesis”	2008	World Journal of Gastroenterology	410	Article
19th	Key et al. [88]	“Diet, nutrition and the prevention of cancer”	2004	Public Health Nutrition	402	Review
20th	Geiser et al. [89]	“Cryptic speciation and recombination in the aflatoxin-producing fungus Aspergillus flavus”	1998	Proceedings of the National Academy of Sciences of the United States of America	396	Article

**Fig. 4:**
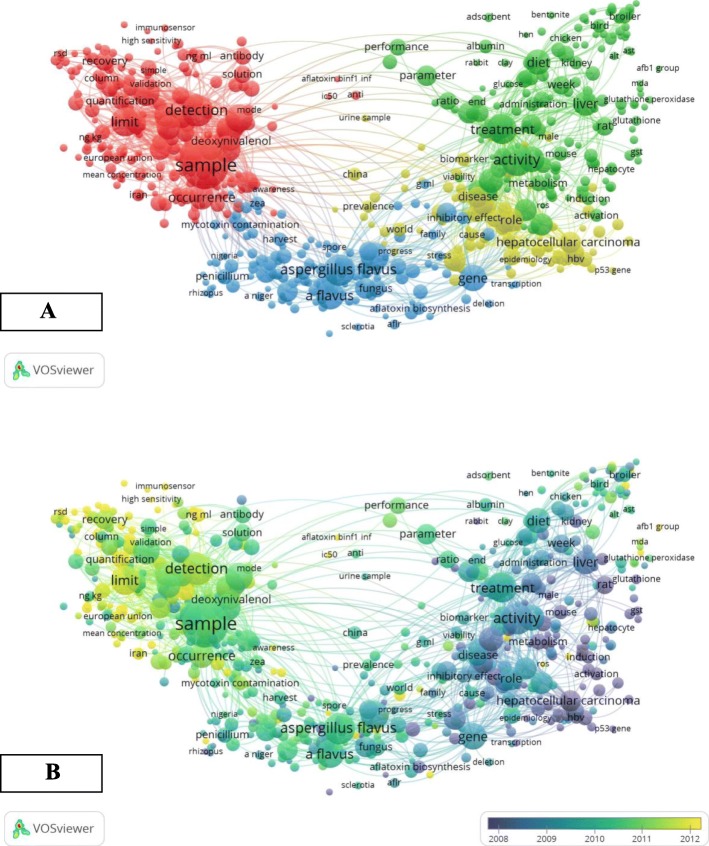
VOSviewer co-occurrence term map of title and abstract words in aflatoxin publications during 1998–2017. **a** The network visualisation term map for aflatoxin research undertaken globally over the 20-year period. **b** Distribution of terms according to the mean frequency of appearance; terms in blue appeared earlier than those in yellow colored terms appeared later

The network visualisation term map for aflatoxin research undertaken globally over the 20-year period from 1998 to 2017 is shown in Fig. 4a. One hundred twenty-eight thousand four hundred twenty different terms were found from the collected publications; however, only 1243 of them appeared more than 40 times. In the term map (Fig. 4a), four thematic research clusters or areas can be noticed, consisting of 1243 co-occurring terms categorising the aflatoxin research field with different four colors. The terms with similarity in research topics are grouped together and the 4 clusters were analyzed as follows:

- Cluster 1 (in red color): this cluster mainly includes the terms related to the topic of detection and quantification of aflatoxin, such as “sample”, “detection”, “solution”; “validation”, “antibody”, “quantification”, “immune sensor”, and “column”.
- Cluster 2 (in blue color): this cluster mainly includes the terms related to the topic of sources and biosynthesis of aflatoxin, such as “*Aspergillus flavus*”, “*A. flavus*”, “spore”, harvest”, “fungus”, “mycotoxin contamination”, and “biosynthesis”.
- Cluster 3 (in yellow color): this cluster mainly includes the terms related to the topic of health effects by aflatoxin, such as “hepatocellular carcinoma”, “disease”, “effect”, “gene”, and “biomarker”.
- Cluster 4 (in green color): this cluster mainly includes the terms related to the topic of detoxification and care regarding aflatoxin, such as “treatment”, “administration”, “diet”, glutathione” and “induction”.

The color of terms was coded by VOSviewer, based on the average time they appeared in the 9845 related publications (Fig. 4b). The blue color indicates the keyword appeared early and red indicates the keywords appeared later. Before 2010, namely in the early stage of research, most aflatoxins’ studies focused on terms related to the topics of “sources and biosynthesis of aflatoxin”, “health effects by aflatoxin”, and “detoxification and treatment of aflatoxin”. The latest trends showed that the terms related to the topic of detection and quantification of aflatoxin would be of concern widely in the future.

One clear theme to emerge from the findings is that the most top-cited aflatoxin publications emphasised the diversity of sub-topics similar to the research hotspots from co-occurring terms including “health effects by aflatoxin” [5, 71, 72, 75, 77, 78, 80, 84–87], “sources and biosynthesis of aflatoxin” [76, 81, 82, 89], “detoxification and treatment of aflatoxin” [74, 79, 88], and “detection and quantification of aflatoxin” [73, 83].

The top ten most prolific institutions in the field of aflatoxin research across the period 1998–2017 are shown in Table 4. *USDA Agricultural Research Service*, of Washington DC, published highest number of aflatoxin publications with 508 documents and covered 5.16% of the total literature. Although the United States led the index, with 6 institutes, there was one institution, respectively, from Argentina, China, Egypt, and Brazil. It is noteworthy that in line with the current findings, previous studies have demonstrated that the USDA is among the bodies with the largest number of works on ecosystem research in several previous studies [92–96].

**Table 4:** The performance of the top 10 most productive institutions in aflatoxin research

Ranking^a^	Institute, country	Number of publications (%)
1st	*USDA Agricultural Research Service, Washington DC, USA*	508 (5.16)
2nd	*United States Department of Agriculture, USA*	404 (4.10)
3rd	*USDA ARS Southern Regional Research Center, USA*	278 (2.82)
4th	*North Carolina State University, USA*	144 (1.46)
5th	*Universidad Nacional de Rio Cuarto, Argentina*	134 (1.36)
6th	*Universidade de Sao Paulo – USP, Brazil*	126 (1.28)
6th	*Texas A and M University, USA*	126 (1.28)
8th	*Chinese Academy of Agricultural Sciences, China*	114 (1.16)
9th	*National Research Centre, Egypt*	110 (1.12)
10th	*Johns Hopkins Bloomberg School of Public Health, USA*	107 (1.09)

^a^Equal institutes have the same ranking number, and then a gap is left in the ranking numbers

standardised

## Limitations

This study utilizes a bibliometric approach to analyze the current status and trend of development of aflatoxin research. But there were a few limitations within which are similar to previous studies. First, the current study was limited by the use of the search term “aflatoxin” in title and/or abstract search only. Particularly, any publications that used “aflatoxin” as a keyword or inside of the publication may have been missed in this analysis. However, if such false-negative results did exist, they will have little effect on the overall findings [7, 35, 38, 39]. Second, it surveyed just the publications in the Scopus database. Although Scopus is the most frequently used and trusted search engine, a few outlier publications might not have been included. Despite that, the current bibliometric study characterises the first concise analysis of the global publications related to aflatoxin by using Scopus and VOSviewer© and illustrates the benefits of bibliometric analysis for assessing research productivity in the field of aflatoxin in a standardised way. Third, the standardization of author names, and terms were completed based on findings on the VOSviewer© and may not be accurate because in certain cases, some authors might have different name spelling or more than one name. This might generate inaccurate research output for these authors. Despite these limitations, this study provides a relatively solid global view on aflatoxin research from these recent two decades.

## Conclusions

The main purpose of this study was to present an overview on the past, present and future scientific research directions of the research field of aflatoxin by combining a bibliometric analysis with a literature review. The quantity of global research output on aflatoxin has substantially increased over the past 20 years, accounting for more than 9800 publications on relevant journals. In earlier years, researchers focused on terms related to the topics of “sources and biosynthesis of aflatoxin”, “health effects by aflatoxin”, and “detoxification and treatment of aflatoxin”. In recent years, researchers paying more attention to the topic of detection and quantification of aflatoxin would be concerned widely with the future. The USA was the largest contributor to aflatoxin scientific research and had the leading position in global research in this field, followed by China. Quite different from other research domains, some developing economies such as India, Iran, Brazil, Turkey, and Egypt were also among the largest contributors. This bibliometric analysis should be of interest to all governmental decisions, healthcare, industries, and educational institutions, involved in the ongoing advances in aflatoxin biosynthesis, better allocation of monitoring efforts, and improved management procedures.

## Supplementary information

**Additional file 1.** Trend of changes in number of publications for aflatoxin research (1963–2018).

## Data Availability

Not applicable.

## Abbreviations

IFs: Impact factors
JCR: Journal Citation Reports
NIH: National Institutes of Health
SNIP: Source Normalized Impact per Paper
WoS: Web of Science
