# Supplementary figures and images for: Global scientific trends on aflatoxin research during 1998–2017: a bibliometric and visualized study
Source: J Occup Med Toxicol. 2019 Nov 21;14:27. doi: 10.1186/s12995-019-0248-7 (PMC6873441; doi:10.1186/s12995-019-0248-7)

**Additional file 1** Trend of changes in number of publications for aflatoxin research (1963-2018).


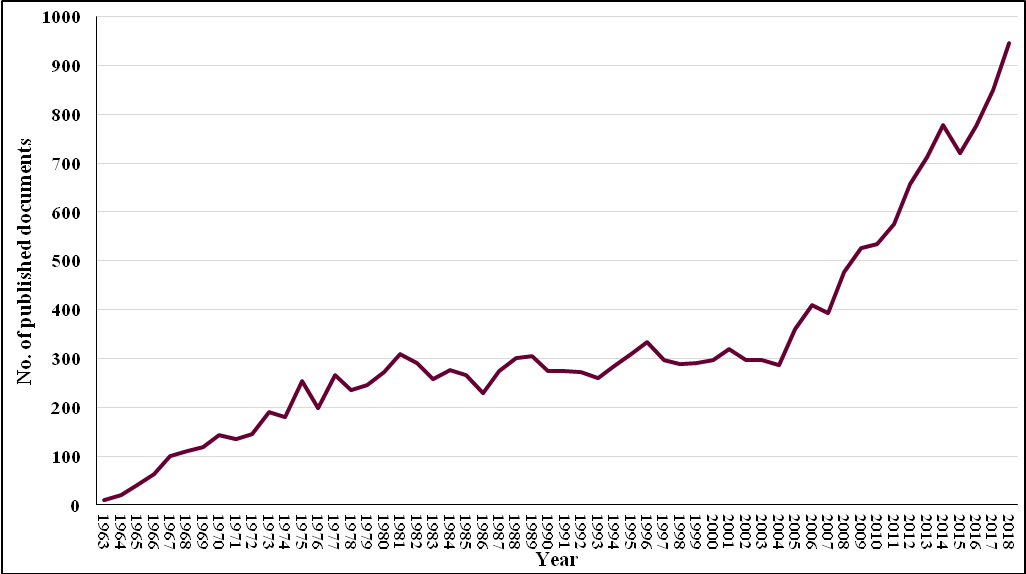

Supplement: Supplementary file 1 — Additional file 1. Trend of changes in number of publications for aflatoxin research (1963–2018). [file 12995_2019_248_MOESM1_ESM.doc]
